# Effect of metformin on the human T98G glioblastoma multiforme cell line

**DOI:** 10.3892/etm.2014.1597

**Published:** 2014-03-04

**Authors:** ALİ UCBEK, ZEYNEP GÜNEŞ ÖZÜNAL, ÖZGE UZUN, AKÇAHAN GEPDİREMEN

**Affiliations:** 1 Department of Pharmacology, Faculty of Medicine, Abant Izzet Baysal University, Bolu 14280, Turkey; 2 Department of Pharmacology, Istanbul Faculty of Medicine, Istanbul University, Istanbul 34390, Turkey

**Keywords:** metformin, T98G, glioblastoma cell line, apoptosis

## Abstract

Metformin is a guanidine derivative found in *Galega officinalis* that is commonly used to treat diabetes mellitus. The mechanism of action of metformin involves regulation of the adenosine monophosphate-activated protein kinase/mammalian target of rapamycin signaling pathway, which is implicated in the control of protein synthesis and cell proliferation. This led to the hypothesis that metformin reduces the risk of cancer and slows tumor growth. Thus, in the present study, the effectiveness of metformin as an antiglioma agent was evaluated using the human T98G glioblastoma multiforme cell line. The viability of the T98G cells was assessed using a 3-(4,5-dimethylthiazol-2-yl)-2,5-diphenyltetrazolium bromide assay. Apoptosis was monitored by measuring caspase-3 levels, as well as by terminal deoxynucleotidyl transferase dUTP nick end labeling and staining with acridine orange and ethidium bromide. The results demonstrate that metformin reduced cell viability and caused apoptotic morphological changes in the T98G cells. Furthermore, the caspase-3 levels in the metformin-treated T98G cells were higher than those in the control cells. Metformin induced apoptosis in the T98G cell line in a concentration-dependent manner. Metformin may provide an important contribution to the treatment of glioblastoma multiforme.

## Introduction

Metformin is widely used for the treatment of type II diabetes; it promotes lower blood glucose levels by increasing muscle glucose uptake, decreasing insulin resistance and improving insulin sensitivity. Metformin has been suggested to be useful for the treatment of diseases other than type II diabetes, including polycystic ovary syndrome and non-alcoholic fatty liver disease, and for reducing the risk of cardiovascular disease (1). Furthermore, a number of studies have shown that metformin plays a role in reducing the risk of certain central nervous system diseases, specifically Parkinson’s and Alzheimer’s diseases (2,3). Studies have increasingly focused on the association between metformin and cancer (4) due to data showing that metformin may reduce the risk of cancer in and improve the prognosis of type II diabetes (5,6). The inhibitory effect of metformin on cancer development and tumor growth is not yet clearly understood, but it may be due to the induction of reductions in systemic glucose and insulin levels (4). Conversely, numerous studies have demonstrated that metformin causes apoptosis and may directly inhibit cell proliferation and induce cell death (7–9). These effects of metformin are explained by its activation of the adenosine monophosphate-activated protein kinase (AMPK)-liver kinase B1 (LKB1) signaling pathway, downregulation of cyclin D1 and inhibition of mammalian target of rapamycin (mTOR) activity (10–13).

Glioblastoma multiforme is the most devastating type of cancer of the central nervous system. The median survival time is generally one year from the time of diagnosis. Despite advances, chemotherapeutics have not been successful due to their high toxicity, limited efficacy and problems with drug delivery (14,15). Novel approaches are required for glioblastoma treatment, including chemotherapy, radiotherapy, and the targeting of apoptosis and cell survival regulatory machinery (16). Given the aforementioned characteristics of metformin, it may be a good candidate for the treatment of glioblastoma. Furthermore, metformin crosses the blood-brain barrier when administered orally and exerts a direct effect on the central nervous system (17).

In the present study, based on epidemiological, clinical and preclinical investigations, the effect of metformin on the human T98G glioblastoma multiforme cell line was examined. The viability of the T98G cells was assessed using a 3-(4,5-dimethylthiazol-2-yl)-2,5-diphenyltetrazolium bromide (MTT) assay. Apoptosis was induced by H_2_O_2_ and monitored by measuring caspase-3 levels, as well as by terminal deoxynucleotidyl transferase dUTP nick end labeling (TUNEL) and acridine orange/ethidium bromide staining.

## Materials and methods

### Cell culture

T98G cells were obtained from Dr Ayhan Bilir, Histology Department, Faculty of Medicine, Istanbul University (Istanbul, Turkey). The T98G glioblastoma cell line was maintained in growth medium [Dulbecco’s modified Eagle’s medium (DMEM)/F12; Gibco-BRL, Carlsbad, CA, USA]. The medium was supplemented with 10% fetal bovine serum (FBS; Gibco-BRL) and 1% antibiotic-antimycotic solution (Gibco-BRL). The cells were incubated in a humidified atmosphere at 37°C and 5% CO_2_. For cell harvesting, 0.05% trypsin-ethylenediamine tetraacetic acid (EDTA; Biological Industries, Kibbutz Beit Haemek, Israel) was used. Metformin (Sigma-Aldrich, St. Louis, MO, USA) was used at concentrations of 1, 5, 10, 50 or 100 mM and H_2_O_2_ (Acros Organics, Fair Lawn, NJ, USA) was used at a concentration of 2.5 mM when applied alone or in combination. Consecutive dilutions were prepared with DMEM/F12 medium.

### MTT assay

The MTT assay is based on the cleavage of yellow tetrazolium salt MTT to purple formazan crystals by metabolically active cells. The MTT test [Cell Proliferation kit I (MTT); Roche Applied Science, Penzberg, Germany] was used to measure cytotoxicity. The T98G cells were seeded in culture plates at a density of 3×10^4^ cells per well in 96-well plates for 24 h. The cells were grown in the 96-well plates in a final volume of 100 μl culture medium supplemented with 10% FBS and 1% antibiotic-antimycotic solution per well. The cells were incubated for 24 h to allow cell adhesion. The total volume was then removed, and the cells were treated with metformin (1, 5, 10, 50 or 100 mM), H_2_O_2_ (2.5 mM), H_2_O_2_ and metformin, or culture medium alone and incubated for 24 h. Following the incubation period, 10 μl MTT labeling reagent (final concentration, 0.5 mg/ml) was added to each well. The 96-well plates were incubated for 4 h. Subsequently, 100 μl solubilization solution was added to each well. The 96-well plates were allowed to stand overnight in an incubator in a humidified atmosphere. Complete solubilization of the purple formazan crystals was then assessed. The 96-well plates were subjected to agitation, and the spectrophotometric absorbance of the samples was run using a (ELISA) microplate reader (Thermo Fisher Scientific, Vantaa, Finland) at 570 nm with a 630-nm reference. The mean absorbance of the control wells served as 100% viability, and the absorbance of sample wells was calculated from the following equation: Viability (%) = optical density in the sample well/optical density in the control well × 100.

### Caspase-3 assay

A caspase-3 colorimetric protease assay (ApoTarget™ Caspase Colorimetric Protease Assay Sampler kit; Novex^®^, Invitrogen Life Technologies, Carlsbad, CA, USA) was performed to detect caspase-3 activity. In this assay, upon cleavage of the substrate by caspase-3, light absorbance by free *p*-nitroanilide (pNA) was quantified using a microplate reader (Thermo Fisher Scientific). The T98G cells were seeded in 25-cm^2^ flasks and incubated for 24 h at 37°C and in 5% CO_2_ to allow cell adhesion. The cells were then treated with metformin (1, 5, 10, 50 or 100 mM), H_2_O_2_ (2.5 mM), H_2_O_2_ and metformin, or culture medium alone, and incubated for 24 h. Following the incubation period, the cells were detached and centrifuged to obtain a pellet. The supernatant was removed, and the cells were resuspended in 50 μl of chilled cell lysis buffer and incubated on ice for 10 min. The tubes were then centrifuged for 1 min in a microcentrifuge (10,000 × g). The cytosolic extract was transferred to a fresh tube and put on ice. The protein concentration was assayed using the biuret method (Biuret solution; Norateks Kimya San. Tic. Ltd. Şti., Istanbul, Turkey). Each cytosolic extract was diluted to an equalized protein concentration with cell lysis buffer. Dithiothreitol (DTT; ApoTarget™ Caspase Colorimetric Protease Assay Sampler kit; Invitrogen Life Technologies) was added to the reaction buffer immediately prior to use. A total of 50 μl 2X reaction buffer containing 10 mM DTT was added to each sample. Subsequently, 5 μl Ac-Asp-Glu-Val-Asp-pNA (DEVD-pNA) substrate was added, and the samples were incubated at 37°C for 2 h in the dark. The absorbance of each sample was read at 405 nm. All samples and controls were run in duplicate. The fold increase in caspase-3 activity was determined by comparison of the absorbance of pNA from the metformin groups with that from the control group.

### Acridine orange/ethidium bromide staining

The T98G cells were seeded in 24-well plates and treated with metformin (1, 5, 10, 50 or 100 mM), H_2_O_2_ (2.5 mM), H_2_O_2_ and metformin, or culture medium alone, and then incubated for 24 h. Subsequently, diluted acridine orange (100 μg/ml; Invitrogen Life Technologies) and ethidium bromide (100 μg/ml; Sigma-Aldrich) in phosphate-buffered saline were added to the wells. The samples and controls were incubated in the dark. After 10 min, the wells were examined by fluorescence microscopy (Olympus CKX41, Olympus U-RFLT50-20, Japan).

### TUNEL assay

The T98G cells were seeded in 24-well plates and treated with metformin (1, 5, 10, 50 or 100 mM), H_2_O_2_ (2.5 mM), H_2_O_2_ and metformin, or culture medium alone, and then incubated for 24 h. Subsequently, labeling of apoptotic cells in the samples was performed using a TUNEL kit (ApopTag; Millipore, Billerica, MA, USA), which involves modifying DNA fragments by utilizing terminal deoxynucleotidyl transferase. Specific staining allowed for the detection of apoptotic cells.

### Statistical analysis

All samples were run at least in triplicate. A one-way analysis of variance was used when multiple comparisons were made. The significance between two groups was determined using Tukey’s test. Data are expressed as the mean ± standard error. P<0.05 was considered to indicate a statistically significant difference.

## Results

### MTT assay

**Figure 1:**
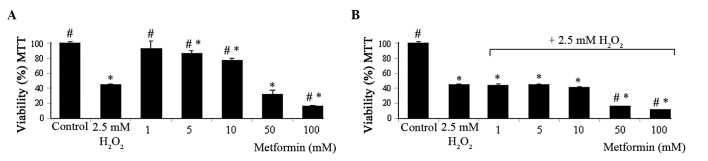
MTT assay viability results in T98G cells. Viability of the T98G cells in the 24 h presence of (A) metformin (1, 5, 10, 50 and 100 mM) and (B) metformin (1, 5, 10, 50 and 100 mM) + 2.5 mM H_2_O_2_ combination. All data are expressed as the mean and SE from three independent experiments. ^*^P<0.001 vs. control, ^#^P<0.001 vs. 2.5 mM H_2_O_2_. MTT, 3-(4,5-dimethylthiazol-2-yl)-2,5-diphenyltetrazolium bromide.

Metformin at concentrations ≥5 mM significantly reduced cell viability compared with that of the control group at 24 h after treatment (Fig. 1A). Metformin concentrations of 10, 50 and 100 mM reduced the cell viability from 100±1.6 to 76.8±3.3, 31.8±5.3 and 16.7±0.7%, respectively. Similar results were observed in the presence of 2.5 mM H_2_O_2_. H_2_O_2_ reduced the viability of the cells by ~50%. In the presence of 2.5 mM H_2_O_2_, metformin reduced the cell viability compared with that of the control group at concentrations ≥1 mM. This reduction was significant at metformin concentrations of 50 and 100 mM compared with the reduction caused by 2.5 mM H_2_O_2_ alone (Fig. 1B).

### Caspase-3 assay

**Figure 2:**
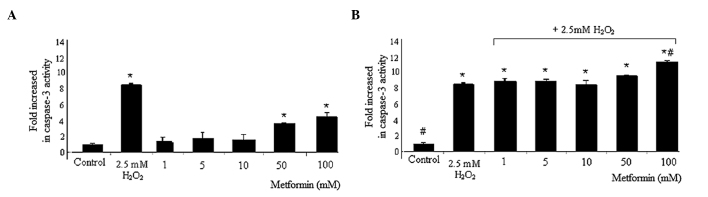
Fold increase in caspase 3 activity in T98G cells. (A) Metformin (1, 5, 10, 50 and 100 mM) and (B) metformin (1, 5, 10, 50 and 100 mM) + 2.5 mM H_2_O_2_ combination. All data are expressed as the mean and SE from three independent experiments. ^*^P<0.001 vs. control, ^#^P<0.001 vs. 2.5 mM H_2_O_2_.

Compared with the caspase-3 levels in the control cells, metformin treatment increased caspase-3 levels significantly at 50 and 100 mM, from 1±0.20 to 3.7±0.1 and 4.9±0.6%, respectively. However, 2.5 mM H_2_O_2_ alone also caused a significant increase in the caspase-3 levels compared with those in the control group (from 1±0.20 to 8.67±0.18%). In the presence of 2.5 mM H_2_O_2_, metformin at 1, 5, 10, 50 and 100 mM increased the levels of caspase-3 from 1±0.20 to 9±0.4, 9±0.2, 8.6±0.5, 9.7±0.1 and 11.5±0.2%, respectively, compared with those in the control group. Additionally, 100 mM metformin + 2.5 mM H_2_O_2_ significantly increased the levels of caspase-3 activity compared with those in the 2.5 mM H_2_O_2_ group (11.5±0.2 vs. 8.67±0.18%, respectively; Fig. 2).

### Acridine orange and ethidium bromide staining

**Figure 3:**
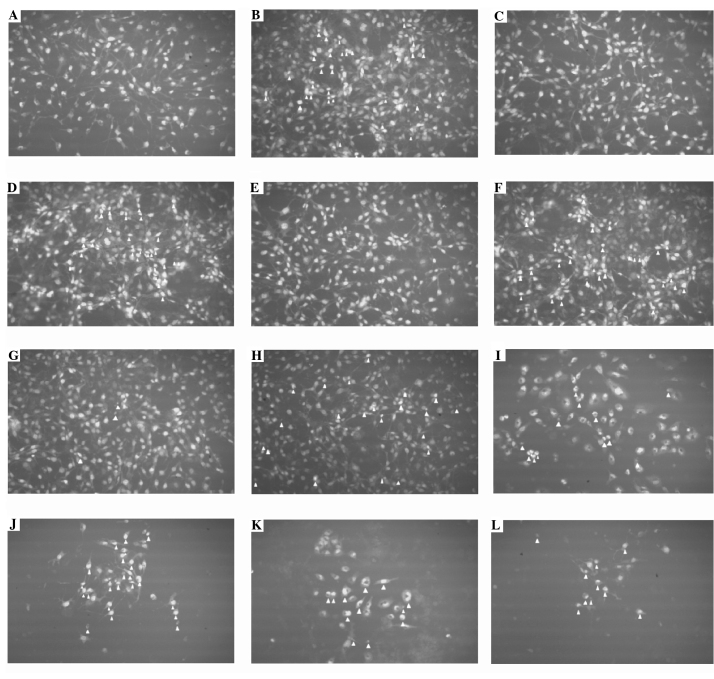
Ethidium bromide and acridine orange fluorescein dye in the T98G cell line (magnification ×400). Apoptotic cells are indicated with an arrowhead. (A) Control, (B) 2.5 mM H_2_O_2_, (C) 1 mM metformin, (D) 1 mM metformin + 2.5 mM H_2_O_2_, (E) 5 mM metformin, (F) 5 mM metformin + 2.5 mM H_2_O_2_, (G) 10 mM metformin, (H) 10 mM metformin + 2.5 mM H_2_O_2_, (I) 50 mM metformin, (J) 50 mM metformin + 2.5 mM H_2_O_2_, (K) 100 mM metformin, (L) 100 mM metformin + 2.5 mM H_2_O_2_.

Apoptotic cells were not observed in the control group. However, apoptotic changes were observed in the presence of 10, 50 and 100 mM metformin and these changes increased as the concentration of metformin increased. Apoptotic changes were also observed following treatment with 2.5 mM H_2_O_2_ alone and with all concentrations of metformin in the presence of 2.5 mM H_2_O_2_. Apoptotic cells are marked with arrows in Fig. 3.

### TUNEL assay

**Figure 4:**
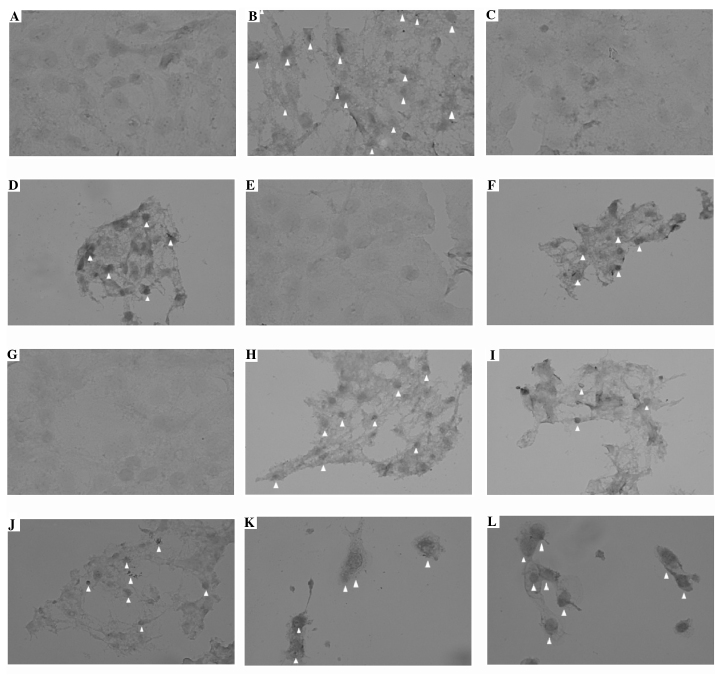
Apoptosis by TUNEL assay of the T98G cell line (magnification ×400). Apoptotic cells are indicated with an arrowhead. (A) Control, (B) 2.5 mM H_2_O_2_, (C) 1 mM metformin, (D) 1 mM metformin + 2.5 mM H_2_O_2_, (E) 5 mM metformin, (F) 5 mM metformin + 2.5 mM H_2_O_2_, (G) 10 mM metformin, (H) 10 mM metformin + 2.5 mM H_2_O_2_, (I) 50 mM metformin, (J) 50 mM metformin + 2.5 mM H_2_O_2_, (K) 100 mM metformin, (L) 100 mM metformin + 2.5 mM H_2_O_2_. TUNEL, terminal deoxynucleotidyl transferase dUTP nick end labeling.

Incorporation of the TUNEL dye into apoptotic cells was assessed 24 h after treatment. Metformin induced apoptotic changes in the 50 and 100 mM groups. Apoptosis-specific morphological changes were observed in the presence of 2.5 mM H_2_O_2_ alone and with all concentrations of metformin. Apoptotic cells are marked with arrows in Fig. 4.

## Discussion

Glioblastoma is the most common and aggressive primary brain tumor of the human central nervous system. The median survival time is approximately one year for patients with advanced tumors. Despite intensive efforts to identify more effective therapies against glioblastoma, the possibility of successful treatment is extremely poor. New approaches have demonstrated that the induction of apoptosis in malignant cells may be a promising strategy in cancer therapy (18). Furthermore, metformin easily crosses the blood-brain barrier (17). In the present study, the effectiveness of metformin as an antiglioma agent was investigated from this perspective. The results show that metformin exerted apoptotic and antiproliferative effects in a concentration-dependent manner, and that these effects were increased in the presence of H_2_O_2_.

In the present study, it was determined using an MTT assay that metformin inhibited the proliferation of the T98G cells, and that the effect was more pronounced following the addition of H_2_O_2_. This effect occurred in a concentration-dependent manner for 1–100 mM metformin. However, it was not possible to use this method to determine how the cells died (19). Thus, different assays were used to determine whether metformin exerts an apoptotic effect.

The nuclear morphology and light emission rates of the T98G cells were evaluated by staining with acridine orange and ethidium bromide. The data showed that apoptotic changes occurred in the cells following treatment with 10, 50 and 100 mM metformin and ≥1 mM metformin in the presence of H_2_O_2_. However, throughout the experiments, cell washing and transfers may have changed the cell population distribution and apoptotic and/or necrotic cell rates (20). The TUNEL and caspase-3 assays in the present study produced similar results for ≥50 mM metformin when used alone. However, as shown in a previous study, metformin-induced cell apoptosis may occur in a caspase-independent manner (7). In the present study, the effects of metformin on apoptosis were observed in the presence of H_2_O_2_. The results indicate that the apoptotic effect of metformin was increased when it was applied in combination with H_2_O_2_.

The combination of metformin with cytotoxic drugs has been shown to markedly inhibit tumor growth (21,22). In this respect, metformin is used as an adjunct to cancer chemotherapy (23,24). However, metformin has been demonstrated to suppress cisplatin-induced apoptosis in cancer cells (25). Although it has been demonstrated in other cell lines, few studies in the literature have reported the apoptotic effect of metformin on glioblastoma cells (26,27). Epithelial growth factor receptor (EGFR) in connection with the phosphoinositol-3 kinase (PI3K) pathway signaling, as well as the association between the EGFR and the mTOR signaling pathway, appears important to maintaining the life of glioblastoma multiforme cells (28,29). Therefore, PI3K and mTOR signaling pathway inhibitors alone or in conjunction with EGFR have become a novel therapeutic target. Metformin as an mTOR inhibitor may provide an important contribution to the treatment of glioblastoma multiforme. The findings of the present study show the effect of metformin on T98G cells and may provide a novel perspective regarding the future treatment of glioblastoma multiforme.

We predict that the direction of future treatment for glioblastoma multiforme will be to optimize surgery to remove the tumor and to use multi-targeted synergistic drug combinations. The targets of the novel drugs are likely to be cell survival mechanisms and apoptosis. In this respect, the apoptotic property of metformin shown in the present study in T98G cells indicate that metformin may be a good candidate for inclusion in treatment protocols.
